# Anæsthetics in Midwifery

**Published:** 1861-12

**Authors:** B. Fordyce Barker


### ANÆSTHETICS IN MIDWIFERY.

At a recent meeting of the New York Academy of Medicine, B. Fordyce Barker, M. D., Professor of Obstetrics in the Bellevue Hospital Medical College, read a paper on the above subject, which elicited the warm commendation of such members of the profession as Drs. Delafield, Peaslee, Gilman, Elliott, Stevens, Detmold, Van Buren and Wooster. The *American Medical Times*, from which we copy, publishes the paper, and we only regret that want of space prevents us from presenting it to our readers, in full—one of the ablest summaries of the views of progressive obstetricians, yet published. The substance, however, we give below; and shall, also, in due time, put our readers in possession of the main points of the discussion the paper has drawn forth among the members of the Academy.

After a spirited résumé and contrast of the diverse opinions held by leading obstetrical authorities, Dr. Barker remarks that the experience of no one individual is sufficient to decide all of the points before alluded to, yet the accumulated observations of all who have had large opportunities will evidently contribute to as fixed principles and rules of practice as can, in the nature of things, be secured in the science of medicine.

In the minds of most medical men the *danger* involved from the use of anæsthetic agents is the grand question above all others. And here permit me to say, that the danger from their use in midwifery, is a question altogether distinct and apart from that of their use in surgery. There has not yet been reported, nor is there any reason for believing that a single death has ever occurred in midwifery practice from the use of *any* anæsthetic agent, where it has been administered by a medical man; and without being able to give statistical evidence in proof of the assertion, I will express my firm conviction, that it has been administered a greater number of times in obstetric than in surgical practice. There are sound and patent physiological reasons why its use should be much less dangerous in the former than in the latter practice.

1st. The conditions under which they are administered, are entirely different. In surgery, the anæsthetic is used to give relief from an *anticipated* suffering. In obstetrics it is used to destroy pain already existing. There is no law better known in medicine than that the tolerance of narcotics and anodynes bears a certain relation to the intensity of the pain. One suffering from peritonitis or colic, can safely, and with advantage, take a quantity of opium, which would be sure to destroy the life of the same individual when in health. For this reason, the risk from such an agent must be very much less in obstetrics than in surgery.

2d. The emotional conditions of the subject under the two circumstances, differ materially; in the one case tending to weaken nerve force and depress the vital powers, and in the other, to secure tolerance of such an agent by stimulating and supporting the same elements. I do not stop here to discuss more fully the influence of the emotions in affecting the vital functions, although, it is a subject of great importance, and one well worthy of the careful study of every practical man. For my present purpose, I think that the mere statement of the proposition is sufficient to secure its acceptance by every mind. When a subject is about to submit to any painful operation and an anæsthetic is proposed, there is always more or less dread and apprehension as to the result, to which is often added an anxiety in regard to the effects of the anæsthetic, whether it will really destroy all consciousness of pain; and if so, whether it will not also destroy life. But in midwifery, the overwhelming desire is to be relieved from the recurrence of the pains, and when the effect of the anæsthetic has once been experienced, it is again sought for with the greatest avidity and confidence.

3d. In midwifery it is ordinarily unnecessary to carry the anæsthetic to the extent to which it is absolutely essential in surgery. In the former, it may frequently be carried to the extent of diminishing or destroying sensation, while consciousness is retained; or, if sleep is induced, it is tranquil not stertorous. But in surgery it is absolutely requisite that the patient be perfectly still, and the anæsthetic must be carried to the extent of complete sopor, the test of which is heavy snoring. Even if it be necessary to carry it to this extent in obstetrical practice, as it may be in some cases of natural labor, and ordinarily when operative measures, either manual or instrumental, are demanded, the two conditions which have been before mentioned as greatly modifying the danger from the anæsthetic still remain. Furthermore, it may be added, that the system is prepared by the previous use of the agent in a less degree, because there is now no emotional resistance to the effect of the anæsthetic. * * *

The propositions which Dr. B. offers as a basis for the discussion of the Academy, are founded on his own clinical experience in 786 cases, in 27 of which, he administered ether, and in the remaining 759, chloroform. Of these, 577 were cases of natural labor, occurring in his private practice. The others are classified under their appropriate heads, and were either cases of difficult labor in his private practice, or in his obstetric service at Bellevue Hospital, or were seen by him in consultation.

In a majority of these cases the chloroform was not carried to the extent of inducing profound anæsthesia. The chloroform was exhibited with the recurrence of pain in such a quantity as to destroy the sensation without overcoming consciousness. The length of time under which patients were kept under its influence, varied from a half hour to, in one instance, over twenty-four hours. In most patients, the inhalations were not commenced until the second stage of labor, but where any special indications existed, it was given any time during the first stage. * * *

The following are the propositions above alluded to, and may be taken, we presume, as embodying the articles of belief of the advocates of anæsthesia in obstetrics:

1st. Anæsthetic aid is of the greatest value in the obstetric art, and chloroform is generally the preferable agent for this purpose.

2d. It exerts no injurious effect, when properly administered, either upon the health of the mother or the child.

3d. It is perfectly justifiable to use chloroform in natural labor, solely for the purpose of relieving pain.

4th. It is especially useful in calming the extreme agitation and mental excitement which labor often produces in very nervous women.

5th. It should be administered in those cases of natural labor where the progress is suspended or much retarded by the pain occasioned by previous diseases, or such as may supervene during labor, and in those cases where the irregular and partial contractions occasion intense and almost constant pain, but have no effect to advance the labor.

6th. It is of great service in spasmodic contraction and rigidity of the cervix uteri, in tetanic rigidity of the perineum, in certain forms of puerperal convulsions, and in the various obstetrical operations.

